# Spontaneous Bilateral Ectopic Pregnancy Treated With Combination of Methotrexate, Unilateral Salpingectomy, and Unilateral Expulsion of Pregnancy

**DOI:** 10.7759/cureus.29031

**Published:** 2022-09-11

**Authors:** Megan Masten, Meredith Alston

**Affiliations:** 1 Obstetrics and Gynecology, University of Colorado, Aurora, USA; 2 Obstetrics and Gynecology, Denver Health and Hospitals, Denver, USA

**Keywords:** diagnostic laparoscopy, ruptured ectopic pregnancy, methotrexate ectopic, bilateral ectopic pregnancy, tubal ectopic pregnancy

## Abstract

Spontaneous bilateral ectopic pregnancies are rare. In the majority of case reports, treatments prescribed were methotrexate, bilateral salpingectomy, or salpingectomy/salpingostomy. A 31-year-old gravida 3 para 0 at our institution underwent diagnostic laparoscopy due to ruptured ectopic pregnancy, and based on visual inspection, had a bilateral ectopic pregnancy. She underwent right salpingectomy for a ruptured ectopic pregnancy and had spontaneous expulsion of the left ectopic pregnancy with mobilization of the fallopian tube. She received methotrexate as per the two-dose protocol and was followed with a negative beta-human chorionic gonadotropin (b-hCG). Pathology confirmed bilateral tubal ectopic pregnancies. Spontaneous bilateral tubal ectopic pregnancy requires a high level of clinical suspicion. If a tubal pregnancy has expulsion of tissue intraoperatively, a two-dose protocol for methotrexate administration may be used for treatment, especially in the case of a bilateral ectopic pregnancy with fertility desires.

## Introduction

The incidence of ectopic pregnancy is estimated to be from 6.4 to 20.7 per 1000 pregnancies based on current studies [1-2]. There are risk factors for ectopic pregnancy such as a history of ectopic pregnancy, infertility, in vitro fertilization, history of sterilization, history of pelvic inflammatory disease, tubal endometriosis, and current intrauterine device use [3-7]. Bilateral ectopic pregnancies are exceedingly rare, with an incidence reported to be 1 in 200,000 pregnancies [8]. A review of this clinical situation analyzed 42 cases over a 10-year period and examined treatment options [9]. Management options depend on multiple factors, including the stability of the patient and future fertility desires. In the reviewed case reports, treatment often consisted of methotrexate, bilateral salpingectomy, or a combination of unilateral salpingectomy and unilateral salpingostomy. There have also been reports of "milking" the fallopian tube to encourage the expulsion of ectopic pregnancy tissue [10-11]. There are no published reports including treatment with unilateral salpingectomy and unilateral expulsion of ectopic pregnancy followed by methotrexate treatment. 

## Case presentation

A 31-year-old gravida 3 para 0 presented to our Early Pregnancy Unit (EPU) with a desired pregnancy. She had a history of a spontaneous miscarriage at six weeks of gestation and a subsequent pregnancy diagnosed with a neural tube defect in the second trimester, with a subsequent dilation and evacuation. Although she had not received any fertility treatment, at an outside institution she had a previous fertility workup with a reported normal hysterosalpingogram.

**Figure 1:**
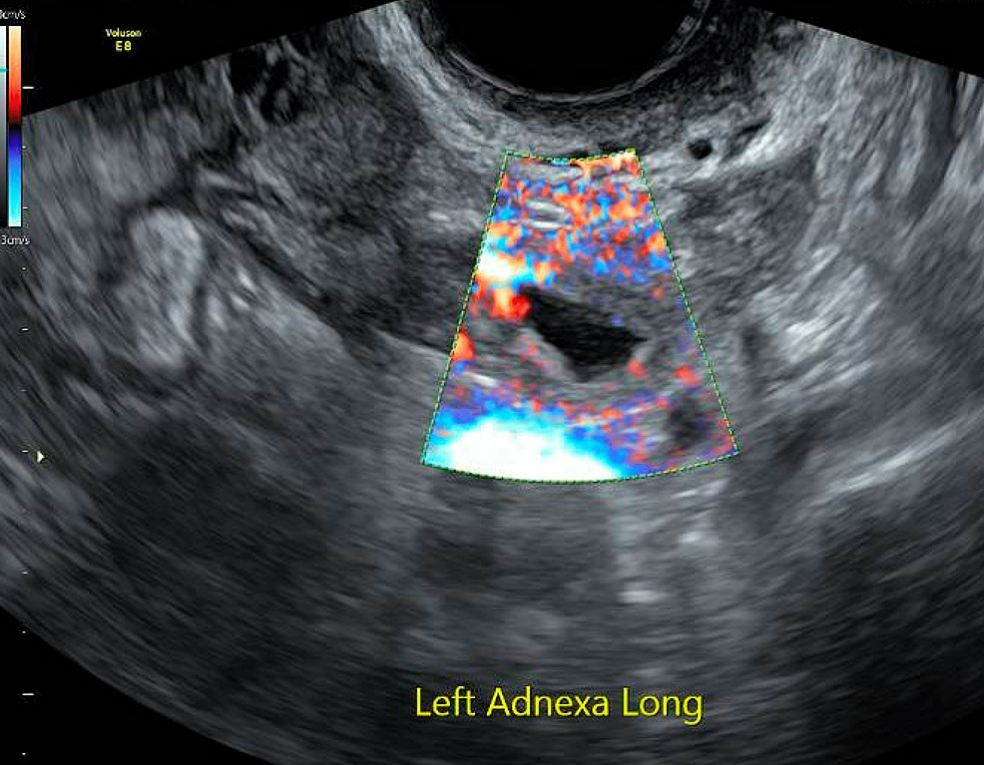
Left adnexal mass on transvaginal ultrasound

**Figure 2:**
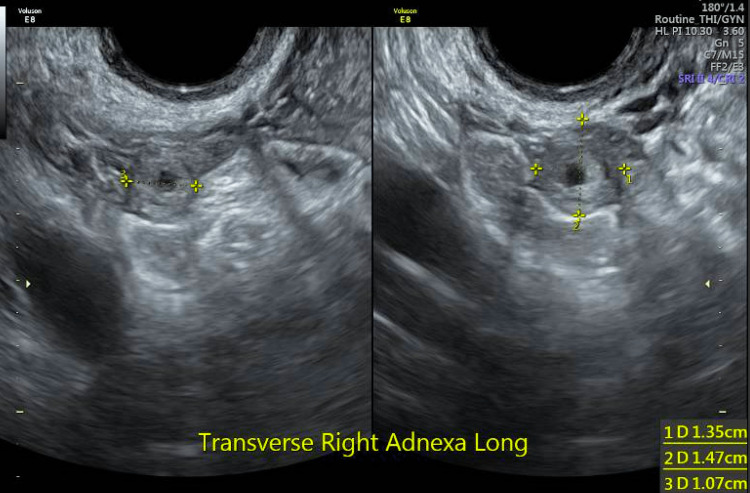
Right adnexal mass on transvaginal ultrasound

She presented to our EPU 6.3 weeks from last menses for vaginal bleeding and pain. On her initial transvaginal ultrasound, she was found to have bilateral solid adnexal masses separate from the ovaries with a pregnancy of unknown location. These bilateral masses had vascular flow in the solid portions, without gestational sacs or fetal poles seen. This was concerning for a spontaneous bilateral ectopic pregnancy versus hematosalpinx or exophytic ovarian masses (Figures 1-2).

At that time, her beta-human chorionic gonadotropin (b-hCG) was 2,793 mIu/mL (milli-international units per milliliter of blood). She had stable vitals and a benign abdominal exam at that time. The patient was monitored closely by our gynecology team for pregnancy at an unknown location and returned 48 hours later for a repeat b-hCG which was 2,359 mIu/mL. At that time, her bleeding and cramping had resolved and with a decreasing b-hCG we recommended presenting in another 48 hours for repeat b-hCG and possible ultrasound. However, the patient presented again later that day with worsening abdominal pain. Her b-hCG was 2,116 mIu/mL and although she was hemodynamically stable with a benign abdominal exam, her ultrasound showed free fluid in the pelvis concerning hemoperitoneum. She was recommended for a diagnostic laparoscopy with possible unilateral or bilateral salpingectomy if there was evidence of a tubal ectopic pregnancy. The patient strongly desired to preserve a fallopian tube if possible for future fertility.

**Figure 3:**
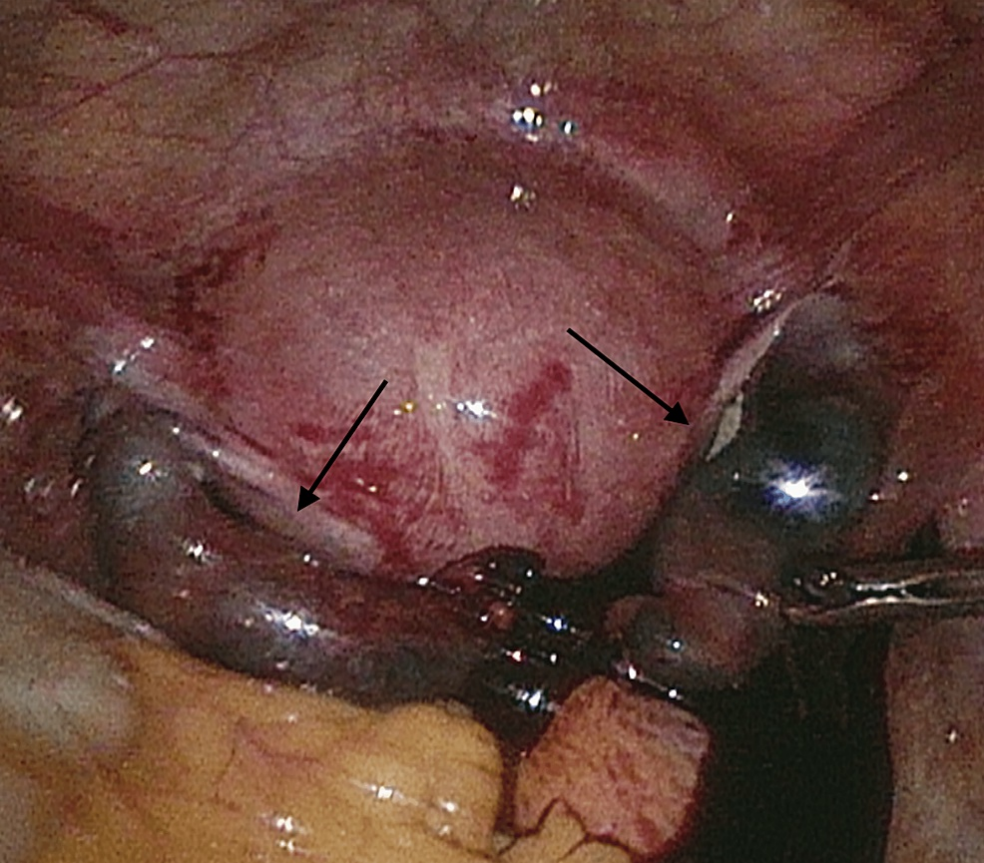
Bilateral ectopic pregnancy with ruptured right fallopian tube

On diagnostic laparoscopy, the patient had bilateral distended, tortuous fallopian tubes (Figure 3). The right fallopian tube was bleeding and consistent with a ruptured right ectopic pregnancy. She underwent a right salpingectomy which was uncomplicated. The left fallopian tube was also distended and tortuous, but when elevated intraoperatively had spontaneous expulsion of hemorrhagic material from the fimbriae into the pelvis. The left fallopian tube was monitored closely for several minutes but it did not bleed, and it appeared that the entire tubal pregnancy was aborted from the fallopian tube. The tubal contents were removed from the abdomen and sent to pathology. She was given a 50 mg/m^2^ dose of intramuscular methotrexate prior to leaving the operating room. She was admitted for one night for serial abdominal exams and serial hemoglobin testing, given concern for possible ongoing bleeding from the left fallopian tube, and both were stable. She was discharged post-operation on day one and returned to the clinic for an additional 50 mg/m^2^ dose of intramuscular methotrexate on day four per the two-dose methotrexate protocol [12].

Her pathology ultimately revealed a right tubal ectopic pregnancy and left fallopian tube contents also consistent with a tubal ectopic pregnancy. She was monitored with b-hCG until negative. She was interested in a future pregnancy and was counseled that there is a high chance of future ectopic pregnancy. She ultimately desired a Reproductive Endocrinology and Infertility referral and is currently undergoing in vitro fertilization.

## Discussion

This was a spontaneous bilateral ectopic pregnancy which was suspected based on ultrasound and diagnostic laparoscopy and confirmed by histopathology. This was a patient not undergoing fertility management, which is exceedingly rare [9]. However, bilateral tubal ectopic pregnancies can happen even in a patient population not utilizing artificial reproductive technologies and a high level of clinical suspicion is required to ensure this diagnosis is not missed. If a patient has ultrasound findings suspicious of a bilateral tubal ectopic pregnancy (for example, bilateral solid adnexal masses separate from the ovary), consider this diagnosis. Despite a falling b-hCG, these patients can still have a rupture of one or both of the tubal ectopic pregnancies. 

This clinical scenario requires unique troubleshooting given the potential that surgical management could leave the patient sterilized. Utilizing alternative management options, including methotrexate therapy, salpingostomy, removal of the pregnancy from the distal opening of the tube, or a combination of measures may be necessary. Additionally, ectopic pregnancies may sometimes be expulsed intraoperatively by "milking" the fallopian tube. In the case of a bilateral ectopic pregnancy in a patient who highly desires future fertility, conservative treatment with methotrexate after spontaneous expulsion of unilateral ectopic pregnancy and a unilateral salpingectomy for a rupturing ectopic pregnancy is reasonable. 

## Conclusions

In the situation that a patient has concern for a bilateral ectopic pregnancy and concern for rupture of one of those pregnancies, a diagnostic laparoscopy must be performed. If there is an obvious ectopic pregnancy with active bleeding and concern for rupture, surgery should be performed with salpingectomy or possibly salpingostomy. However, if the tubal ectopic pregnancy tissue has been expulsed from the other fallopian tube, it may be safe to treat the patient with methotrexate if the patient desires future fertility. 
